# Aortic dissection extending from the brachiocephalic artery during transradial coronary catheterization: a case report

**DOI:** 10.1186/s12872-020-01687-8

**Published:** 2020-08-31

**Authors:** Kihyun Kim, Yeon Seong Kim, Yeongmin Woo, Sang-Yong Yoo

**Affiliations:** Department of Cardiology, Gangneung Asan Hospital, University of Ulsan College of Medicine, 38 Bangdong-gil, Sacheon-myeon, Gangneung-si, Gangwon-do 25440 Republic of Korea

**Keywords:** Iatrogenic aortic dissection, Transradial catheterization, Brachiocephalic trunk, Conservative treatment, Case report

## Abstract

**Background:**

Iatrogenic acute aortic dissection (AD) is an extremely rare but devastating complication during cardiac catheterization. It can be treated conservatively if it develops in a retrograde form or manifests as an intramural hematoma (IMH) with a micro-intimal tear in the absence of instability. However, only a few reports exist on its natural course and long-term outcomes.

**Case presentation:**

A 78-year-old woman presented to the emergency department with acute chest discomfort. Elective cardiac catheterization was performed via the right radial artery. The patient’s brachiocephalic artery was so tortuous that the hydrophilic soft guidewire had to be exchanged for a stiffer one. However, the stiff wire caused the dissection of a tortuous brachiocephalic artery that extended from the sinuses of Valsalva to the proximal descending aorta. Emergent computed tomography showed crescentic aortic wall thickening without a dissection flap. The patient had cardiac tamponade and a gradually thickening thrombosed false lumen. Although the patient was unstable during the first 2 weeks, she was stabilized during hospital stay with only conservative treatment. Consequently, she has been well for over 5 years.

**Conclusions:**

Even though the patient showed ominous findings, a good prognosis was expected because the AD was mainly retrograde. Furthermore, the thrombosed false lumen mimicked an IMH on imaging. To the best of our knowledge, this is the first report of an extensive iatrogenic AD originating from the brachiocephalic artery during right transradial catheterization that was treated conservatively despite clinical instability.

## Background

Acute iatrogenic aortic dissection (AD) is an extremely rare but potentially lethal complication during cardiac catheterization. Only a few studies have been conducted on its natural history and long-term outcomes. Previous case series have suggested that iatrogenic ADs extending > 40 mm above the ascending aorta should be treated surgically. However, recent studies have reported that iatrogenic AD can be treated conservatively if it develops in a retrograde form or manifests as an intramural hematoma (IMH) with a micro-intimal tear. Here, we report an extremely rare case of an acute DeBakey Type I AD that occurred during right transradial cardiac catheterization. Despite hypotension and increasing IMH thickness, the patient was successfully treated without surgery.

## Case presentation

**Fig. 1:**
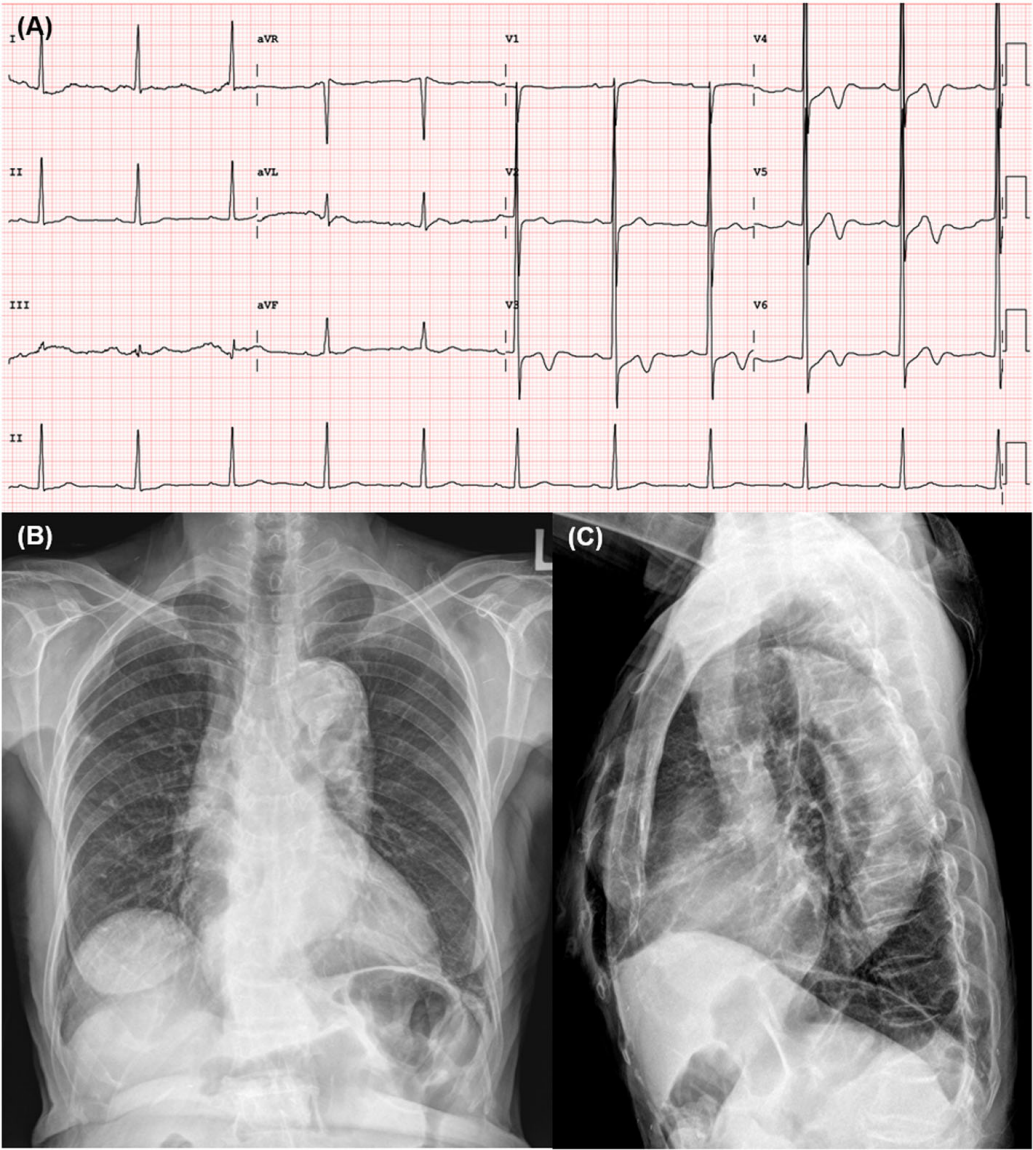
Electrocardiogram (ECG) and chest X-rays before the catheterization procedure **a** ECG shows biphasic T wave inversions in leads V3–V6 and left ventricular hypertrophy without interval change; **b** and **c** Chest X-rays reveal cardiomegaly and a tortuous, calcified aorta

**Fig. 2:**
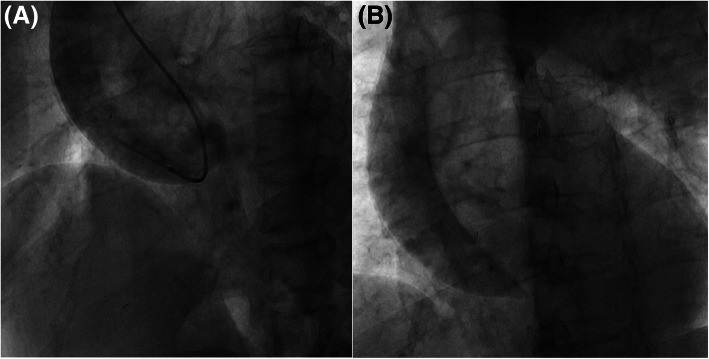
Cardiac catheterization performed via right radial access. **a** After passage of the stiff wire through the tortuous brachiocephalic artery, the first injection of contrast dye in the aortic root reveals a discrete dissection flap from the sinus of Valsalva to the origin of the brachiocephalic artery; **b** The final angiogram shows persistent dye staining of the dissection flap


A 78-year-old woman with a history of hypertension and apical hypertrophic cardiomyopathy presented to the emergency department with a 30-min history of atypical chest discomfort and dizziness. She was taking bisoprolol 2.5 mg qd, lercanidipine 20 mg qd, valsartan 160 mg qd, spironolactone 12.5 mg qd, and torsemide 5 mg qd for hypertension. She had no history of cigarette smoking, alcohol intake, or connective tissue disease and no family history of acute aortic syndrome. Her electrocardiogram (ECG) showed biphasic T wave inversions in leads V3–V6 and left ventricular hypertrophy (Fig. 1a). Biphasic T wave inversions in anterolateral leads could be due to apical hypertrophic cardiomyopathy as there was no interval change compared to a previous ECG, but the possibility of ischemic change could not be completely excluded. Chest X-ray revealed cardiomegaly and a tortuous and calcified aorta (Fig. 1b and c). Echocardiogram showed normal left ventricular ejection fraction (LVEF) without regional wall motion abnormality (Additional file 1 and 2: Video S1–2).. Thus, Elective cardiac catheterization was performed via the right radial artery. Only aspirin was administered from 3 days before cardiac catheterization and 3000 units of heparin were administered after radial artery puncture on the day of cardiac catheterization. The patient’s brachiocephalic artery was so tortuous that the 0.035″ hydrophilic soft guidewire had to be exchanged for a stiffer one. A 5-Fr Judkins right catheter was then introduced into the ascending aorta. The first contrast dye injection into the aortic root made a discrete dissection flap from the sinus of Valsalva to the origin of the brachiocephalic artery (Fig. 2a and Additional file 3: Video S3). Persistent dye staining within the dissection flap was observed on the final angiogram (Fig. 2b and Additional file 4: Video S4). Thus, the procedure was discontinued without coronary angiogram.

**Additional file 3: Video S3.** Fluoroscopic anteroposterior view of the ascending aorta. The first contrast dye injection into the aortic root made a discrete dissection flap from the sinus of Valsalva to the origin of the brachiocephalic artery


**Additional file 4: Video S4.** Rotational fluoroscopy of the ascending aorta which shows persistent dye staining within the dissection flap


**Additional file 5: Video S5.** Axial contrast-enhanced computed tomography (CT) shows moderate amounts of pericardial effusion and crescentic aortic wall thickening without a dissection flap. The site of entry tear is suspected to be in the proximal brachiocephalic artery. This aortic dissection (AD) mimics an intramural hematoma (IMH) extending bidirectionally.

**Fig. 3:**
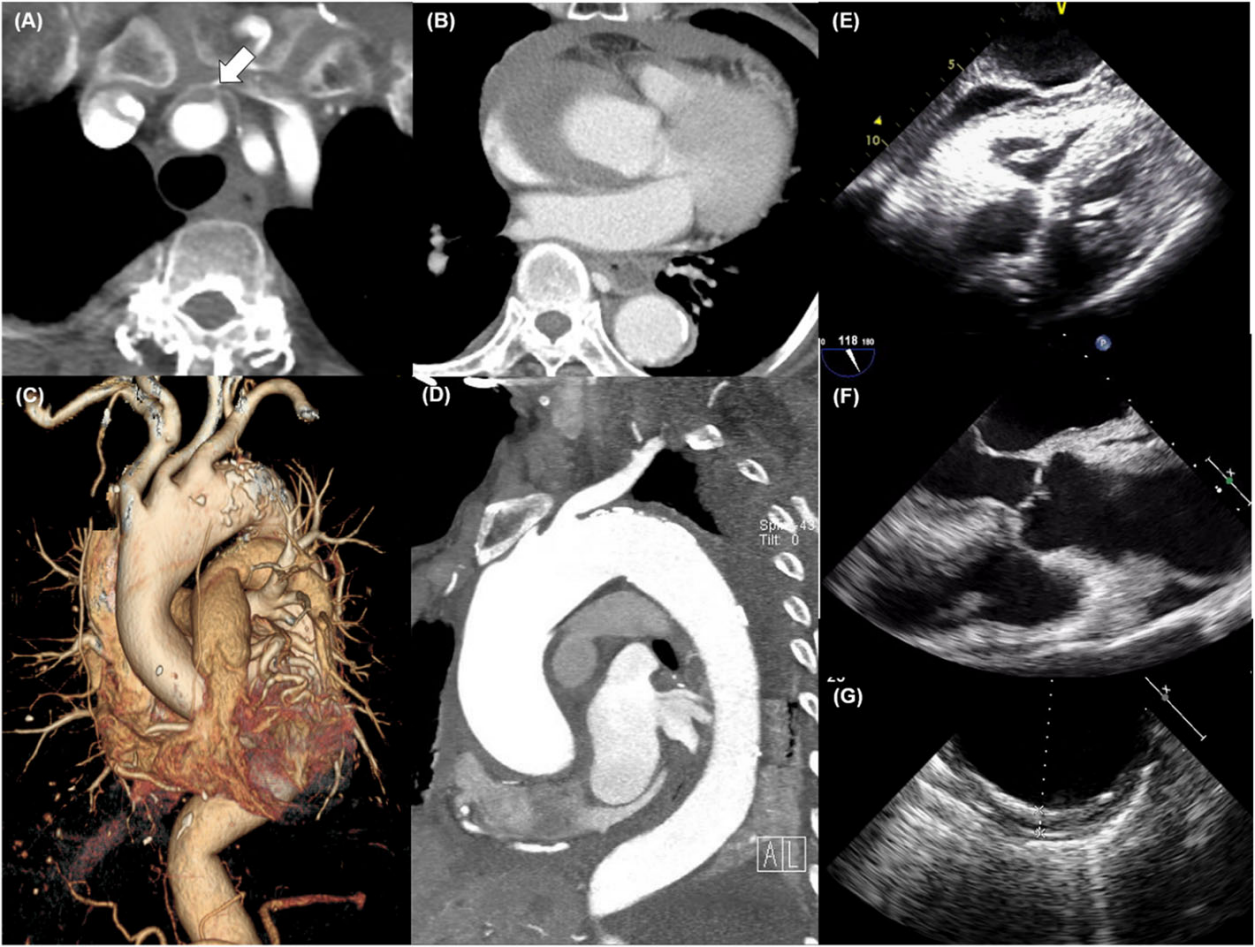
**a**-**d** Computed tomography (CT) angiography and **e** transthoracic and **f**-**g** transesophageal echocardiography (TEE) scans; **a**-**b** Aorta CT angiography, axial plane. **a** The site of entry tear is suspected to be located in the proximal brachiocephalic artery (white arrow) but a dissection flap cannot be clearly observed; **b** This aortic dissection (AD) mimics an intramural hematoma (IMH) retrogradely extending to the sinuses of Valsalva resulting in pericardial effusion; **c** Three-dimensional reconstructed image and **d** Sagittal oblique reconstruction image of aorta CT angiography shows the extent of AD mimicking an IMH extending anterogradely to the proximal descending thoracic aorta; e subcostal view of transthoracic echocardiogram reveals newly developed, moderate amount of pericardial effusion; f TEE (135 degree long axis view) showed no definite dissection flap and intramural hematoma in the aortic root. g TEE (0 degree short axis view) revealed an IMH extending anterogradely to the proximal descending thoracic aorta

The patient’s vital signs were stable, and there was no evidence of pericardial effusion on immediate echocardiography. Five hours later, however, the patient complained of chest discomfort and back pain. Her blood pressure (BP) dropped to 85/50 mmHg, and her pulse rate was 48 bpm. Emergent echocardiography and computed tomography (CT) showed moderate amounts of pericardial effusion and crescentic aortic wall thickening without a dissection flap (Fig. 3a-g). The site of entry tear was suspected to be in the proximal brachiocephalic artery (Fig. 3a and Additional file 5: Video S5). These findings suggested an AD with a thrombosed lumen mimicking IMH. The AD extended in both directions: retrogradely, to the sinuses of Valsalva (Fig. 3b) and anterogradely, to the proximal descending thoracic aorta (Fig. 3c-d, g). Blood flow to the branches of the aortic arch was preserved (Fig. 3 c-d).

**Fig. 4:**
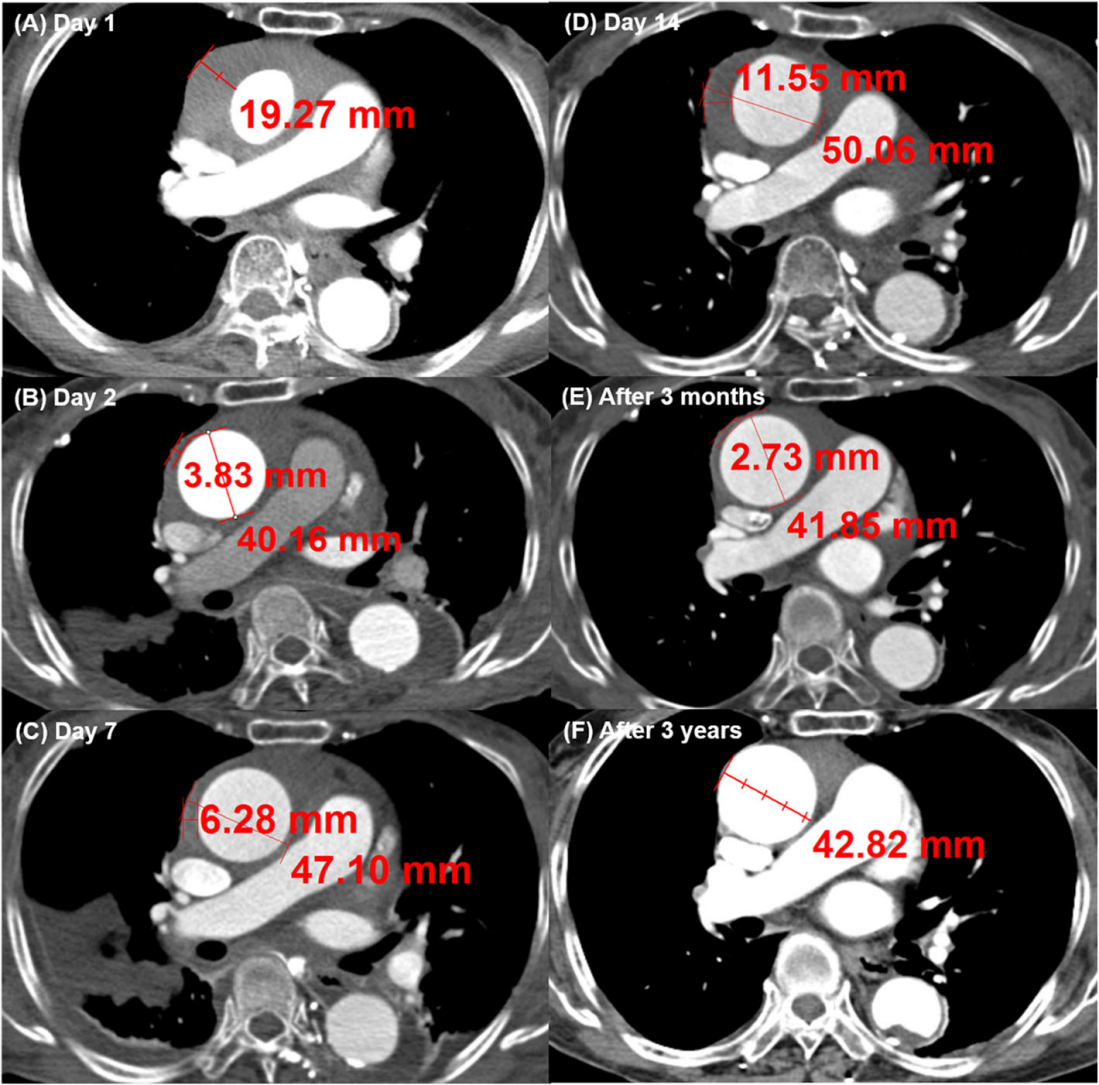
Axial contrast-enhanced computed tomography (CT) showing the change in intramural hematoma (IMH) thickness at the mid-ascending aorta. **a**-**b** The day after the occurrence of the catheter-induced aortic dissection, the IMH thickness is noted to decrease from 19 to 4 mm by conservative treatment; **c**-**d** However, it gradually increases to 11 mm until the second week. The diameter of the ascending aorta is 50 mm; **e**-**f** After 3 months, the IMH thickness has decreased to 2.7 mm. Follow-up CT after 3 years shows complete resolution of the IMH

Her BP stabilized temporarily with rapid infusion of normal saline and 6% hydroxyethyl starch, but soon fell again. So we decided on emergency surgery for the acute AD, and at her request, she was immediately transferred to Asan Medical Center, Korea’s largest referral hospital. However, her BP did not drop again and chest discomfort and back pain also improved. Furthermore, follow-up CT, taken 5 h after the first CT showed a significant decrease in the IMH thickness from 19 to 4 mm at the mid-ascending aorta (Fig. 4a-b). Follow-up echocardiogram revealed normal LVEF with small amount of pericardial effusion and mild aortic regurgitation. Therefore, the surgery was withheld and she was monitored closely in the intensive care unit. For the first 3 days, intravenous esmolol was administered to control BP and pulse rate, and then changed to bisoprolol. Timeline of relevant events were presented in Table 1.

**Table 1:** Timeline of relevant events

Date	Time	Events
07-Apr-2015		Onset of chest discomfort
07-Apr-2015		First hospitalization
14-Apr-2015	15:00	Elective cardiac catheterization
	20:30	BP drop with chest discomfort and back pain
	21:50	Emergent aorta CT angiography
	23:20	Decided to transfer to the referral hospital for emergency surgery
15-Apr-2015	02:40	Follow-up aorta CT angiography in the referral hospital
07-May-2015		Discharged from the referral hospital

However, thereafter, the IMH thickness gradually increased to 11 mm until the second week (Fig. 4c-d). In the third week, there was no further increase in IMH thickness on follow-up CT. The patient remained stable and was discharged 3 weeks after hospitalization without surgery. She was prescribed bisoprolol 7.5 mg qd, amlodipine 5 mg qd, olmesartan 10 mg qd, and spironolactone 12.5 mg bid as discharge medications. After 3 months, the IMH thickness decreased to 2.7 mm, and it disappeared completely after 3 years. The diameter of the ascending aorta remained at 50 mm (Fig. 4e-f). Five years later, the patient remains well with medical treatment.

## Discussion and conclusions

Acute iatrogenic AD is an extremely rare (incidence: 0.01–0.02%) yet devastating complication during cardiac catheterization [1]. It may be classified into two main types: anterograde and retrograde. The former type can be treated with a stent, while the latter usually seals spontaneously with the collaboration of antegrade blood flow [2, 3]. Due to the rarity of iatrogenic AD, only a few reports exist on its natural course and long-term outcomes [4].

In 2000, Dunning et al. reported nine cases of iatrogenic aortocoronary dissection and proposed a classification involving three grades [5]. Two cases were classified as class 3; both these patients underwent emergency surgery and died. Thus, it was recommended that surgical intervention be performed for class 3 dissections involving the cusp and extending > 40 mm above the ascending aorta.

In contrast, a recent study involving 74 cases and a median follow-up of 5 years reported that patients with iatrogenic AD involving ascending aorta had a good prognosis after the acute phase, regardless of coronary artery involvement and vascular access (femoral or radial) [6]. Only two patients with ischemic heart disease died of cardiogenic shock within a month of follow-up.

In most cases, iatrogenic AD occurring during cardiac catheterization is caused by mechanical trauma from a catheter, a guidewire, or other devices around an ostium of a coronary artery. There have been only two case reports of iatrogenic AD in which the intimal flap was reported to be located in the brachiocephalic artery. One patient underwent surgery [7] while the other was treated medically because of the absence of instability [8].

To the best of our knowledge, this is the first report of an extensive iatrogenic AD originating from the brachiocephalic artery during right transradial catheterization that was treated conservatively despite clinical instability. This was possible because the patient was re-stabilized while the patient was going to the referral hospital, which is 3 h away by car to receive emergency surgery, and the aortic dissection was greatly reduced in the follow-up CT scan. Hemorrhage into the pericardium could have resulted in cardiac tamponade. If the patient had not been transferred, she would have received an emergency surgery immediately because she was unstable at the beginning with also a pericardial effusion, which is a very important prognostic factor. For reference, the author’s hospital is a referral hospital with 850 beds, 8 interventional cardiologists and 2 catheterization labs. We perform about 1300 coronary angiograms and 800 percutaneous coronary interventions annually.

The risk of iatrogenic dissection increased by inadvertent and aggressive manipulation of a wire or a catheter in heavily calcified vessels. Even though the patient showed ominous findings such as aggravated back pain, newly developed pericardial, and hypotension, a good prognosis could be possible because the AD was mainly retrograde. Furthermore, the thrombosed false lumen mimicked an IMH on imaging [2].

An iatrogenic AD may occur in the form of an IMH with a micro-intimal tear that cannot be easily detected on CT or transesophageal echocardiography [9]. For such variant forms of AD, imaging findings are more important than clinical features. Interestingly, in Eastern countries, a type A IMH was more prevalent than classic AD (25.5% vs. 10.9%). AD was managed by medical treatment more frequently (80.8% vs. 48.8%) and was associated with lower hospital mortality (5.9% vs. 33.3%) in Eastern countries than in Western countries [10].

To prevent such terrible complications, the operator should not manipulate the wire or catheter forcefully when resistance is encountered. It is a good practice to check the pressure before test injection of the contrast dye. Particularly, on encountering a tortuous vascular access, the operator should consider trying other routes of vascular access with a lower risk of dissection. If complications occur, it is important to discuss appropriate treatment strategies with other cardiologists, radiologists, and cardiac surgeons.

In the clinical practice of catheterization, operators should handle guidewires and catheters carefully to prevent potentially life-threatening complications. If an iatrogenic AD occurs, careful examination of imaging findings, an understanding of the mechanism of injury, vigilant monitoring of the patient’s clinical course, and identification of the best treatment strategy are warranted.

## Supplementary information

**Additional file 1: Video S1.** Parasternal long axis view of transthoracic echocardiogram showed apical hypertrophic cardiomyopathy with normal left ventricular ejection fraction (LVEF).

**Additional file 2: Video S2.** Apical 2-chmabers view of transthoracic echocardiogram showed apical hypertrophic cardiomyopathy with normal LVEF.

## Data Availability

All data generated or analysed during this study are included in this published article and its supplementary information files.

## Abbreviations

AD: Aortic dissection
IMH: Intramural hematoma;
ECG: Electrocardiogram
CT: Computed tomography
LVEF: Left ventricular ejection fraction
